# Sound Absorption Performance of the Poplar Seed Fiber/PCL Composite Materials

**DOI:** 10.3390/ma13061465

**Published:** 2020-03-23

**Authors:** Yingjie Liu, Lihua Lyu, Jing Guo, Ying Wang

**Affiliations:** School of Textile and Material Engineering, Dalian Polytechnic University, Dalian 116034, China; 18840928558@163.com (Y.L.); guojing@dlpu.edu.cn (J.G.)

**Keywords:** poplar seed fibers, polycaprolactone, composite material, sound-absorption properties, fractal dimension

## Abstract

Composite materials were prepared by the hot pressing method using poplar seed fibers and polycaprolactone (PCL) as the raw materials to solve the problems related to the recycling of waste fibers. The effects of mass fraction of poplar seed fibers, the volume density, and thickness on the sound absorption performance of the resulting composite materials were studied. The sound absorption coefficient curves of the composite material were obtained by the acoustic impedance transfer function method. The sound absorption coefficient of the composite material that was prepared under the optimal process conditions was higher than 0.7, and the effective sound absorption frequency band was wide. According to the box counting dimension method, which is based on the fractal theory, the fractal dimensions of the composite materials were calculated while using the Matlab program. The relationships between the fractal dimensions and the volume densities, mass fractions of poplar seed fibers, and thicknesses of the composite materials were also analyzed. Subsequently, the quantitative relationship between the fractal dimension and the sound absorption property parameters of the composite material was established in order to provide a theoretical basis for the design of the sound absorption composite material.

## 1. Introduction

Strengthening greening construction has become a widespread concern with the enhancement of people's awareness of ecological environment protection. Poplar is widely planted as the main tree species to strengthen environmental greening due to its advantages. Up to 2008, the total area of poplar forest in China is over 70,000 km^2^, ranking first in the world and increasing year-by-year. Each poplar tree can produce poplar seed fibers about 25 kg [1,2]. Therefore, every spring, people will find a large number of poplar seed fibers falling down. Poplar seed fibers float around and easily enter the human body through nasal cavity and oral cavity, resulting in allergy of susceptible people, and in serious cases, respiratory system diseases, liver and kidney injuries, heart failure, and so on [3]. The fiber surface also carries dust and bacteria, which will pollute the water source if it falls into the water body. Moreover, flying poplar seed fibers are easy to cause fire and pose a threat to people's lives [4]. Presently, the harm of poplar seed fibers has been widely concerned, which has become one of the factors affecting the construction of livable cities, and it is also a major problem that the landscaping department must face [5]. At the same time, poplar seed fiber is a kind of municipal solid waste. At present, most of its treatment methods are burying and burning, which will not only cause serious pollution to the natural environment, but also a waste of natural organic resources. Therefore, it has become a substantive issue for every scientific research worker to properly handle the poplar seed fibers, prevent it from being abandoned in nature, and reduce the environmental burden.

Poplar seed fibers have excellent properties, such as fast moisture absorption and moisture conduction, good warmth retention, fluffiness and elastic recovery, thin fiber wall, small thermal conductivity, good thermal insulation, large hollowness (the radial hollow area accounts for 80–90% of the cross-sectional area), light weight, water repellent, and oil absorbing property [6], which has application value in many fields. At present, the application of poplar seed fibers is mostly concentrated in the fields of thermally insulating materials [7], oil absorbing materials [8], carbon nanotubes [9], adsorbents [10], capture microprobes [11], and carbon aerogels [12]. The preparation of thermal insulation materials from poplar seed fibers needs improvement in fiber impurity removal and floc forming. Oil absorption materials that are prepared from poplar seed fibers still need to be strengthened in terms of pat uniformity control. The preparation of carbon microtubules, adsorbents, captured microprobes, and carbon aerogels from poplar seed fibers has complicated preparation process, high processing cost, and incomplete utilization of poplar seed fibers.

Recently, researchers have made increasing kinds of sound absorption materials. Fiber reinforced materials have broad application prospects due to their environment-friendly and sustainable development characteristics. For fiber porous sound absorbing materials, the structure has the following characteristics: (1) there are a large number of micropores or gaps uniformly distributed in the material. (2) The micropores in the material are connected with each other, and the sound absorption effect cannot be achieved by single bubbles and closed gaps. (3) The micropores in the material are opened outwards, so that sound waves can easily enter the micropores and good sound absorption performance can be obtained. Jiang et al. [13] combined barium titanate/nitrile rubber (BT/NBR) and polyurethane foam in order to prepare sound absorbing material with alternating multilayer structure. The results show that the BT/NBR-PU foam composites with alternating multilayered structure have am excellent sound absorption property at low frequency due to the organic combination of airflow resistivity, resonance absorption, and interface dissipation. Ahmad et al. [14] prepared the kenaf/bamboo fiber reinforced epoxy hybrid composite by the manual lamination method, and improved the sound absorption performance of the composite by introducing an air gap. It shows that increasing the porosity of the composite material is of great help in the improvement of the sound absorption capability of the material. Han et al. [15] investigated the structural characteristics of kapok fibers. The results showed that the air layer sealed by the hollow cavity of kapok fiber created the natural advantage of low density and sound insulation. Shen et al. [16] made use of the highly hollow property of kapok fiber to prepare sound-absorption composite materials with excellent sound absorption performance, which showed the application prospect of hollow fiber in the field of sound absorption and noise reduction. Our research group found, through experiments, that poplar seed fibers had low density, high hollowness, regular structure, plasticity, easy molding, easy curling, and winding together to form a large number of interconnected pores, which was expected to be more widely and deeply applied in the field of sound absorption field. 

A fractal is defined as a mathematical object with a fractional (non-integer) dimension. The inside of a nonwoven fabric [17], a fiber-reinforced composite material [18,19], a fiber aggregate [20], or a similar substance is characterized by a porous body that was composed of fibers and pores. The pore shapes are not regular or smooth, the pore sizes are also different, and there is no characteristic dimension that can indicate the internal pore structure [21]. Fractal geometry is a new tool for simulating irregular pore structures of materials. The relationship between the fractal dimension and the structural parameters of porous materials can be obtained through fractal image processing and mathematical calculations. Poplar seed fiber/PCL composite materials belong to heterogeneous and multiphase composites in terms of microscopic size and phase state. It is a complex and irregular porous material that is formed by bonding poplar seed fibers through PCL resin, thus forming fiber contact points, fiber segments between contact points, and pores that formed between fibers. Fractal theory can solve the disordered and irregular complex system, and it can accurately and quantitatively express its internal complex structure, thus establishing the relationship between it and sound absorption performance. 

In order to provide a new research direction for the recovery and utilization of poplar seed fiber and provide experimental basis and theoretical guidance for the design of sound absorption composite materials with high sound absorption coefficient, wide frequency band, and wide application range, poplar seed fiber and polycaprolactone are used as raw materials for preparing composite materials by the hot pressing method, which can fully utilize the poplar seed fiber. The effects of poplar seed fiber mass fraction, material density, and material thickness on the sound absorption performance of the composite were analyzed in detail. The relationship between fractal dimension and average sound absorption coefficient, fractal dimension, and resonance sound absorption frequency was quantitatively described by the box counting method.

## 2. Experiment

### 2.1. Materials and Equipment

Poplar seed fibers with an average length of 4 ± 1 mm and density of 0.360 g/cm^3^ (Huang Fan District Farm, Henan, China) and Polycaprolacton powder with a density of 1.021 g/cm^3^ and a melting point 60 °C (Suzhou Yifu Plasticizing Co., Ltd., Taicang, China) were used as the raw materials. An Shanghai Jingke Analytical Balance (MP2000D, Tianma Instrument Factory, Tianjing, China), a hot pressing machine (QLB-50D/Q, Zhongkai Rubber Machinery Co., Ltd., Wuxi, China), an impedance-tube sound-absorption test system (SW422/SW477, Shengwang company, Beijing, China), and an scanning electron microscope (JEOL JSM-6460LV, JEOL Ltd., Beijing, China) were used for testing.

### 2.2. Preparation of the Composite Materials 

Through the hot pressing method, the poplar seed fiber/PCL composite materials were prepared while using poplar seed fibers and PCL as raw materials. The poplar seed fibers and PCL were mixed in a certain proportion and heated to 90 °C at 10 MPa for 20 min. using hot pressing machine. Subsequently, the composite materials were molded and cylindrical test samples having a bottom surface diameter of 100 mm and heights of 10 mm, 15 mm, 20 mm, 25 mm, and 30 mm, respectively, were prepared.

### 2.3. Testing of the Composite Materials

#### 2.3.1. Testing of the Sound Absorption Coefficient

**Figure 1:**
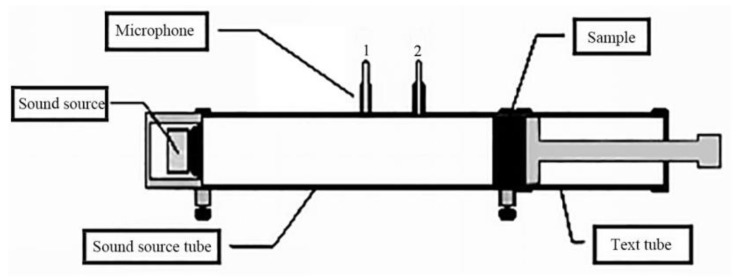
Schematic diagram of the sound-absorption test.

The test standard for sound absorption coefficient of composite materials was GB/T 18696.2-2002 and GB/T 18696.1-2004. Under the conditions of atmospheric temperature (24 °C), the velocity of sound wave (345.60 m/s), a characteristic impedance of air of 409.80 Pa·s/m, and a relative humidity of 65%, the sound absorption coefficient of the samples at different frequencies were tested using SW422/SW477 impedance tube sound absorption test system [22]. The measured sound absorption coefficient was the average of six measurements, and then the sound absorption coefficient curves of materials under different frequencies were drawn. Figure 1 shows the sound absorption coefficient curve test chart.

For materials, when the average sound absorption coefficient is greater than 0.20, it is called sound absorption material, and it is called high-efficiency sound-absorption material when it is greater than 0.56 [23]. The average sound-absorption coefficient refers to the average value of the sound absorption coefficient at frequencies of 125 Hz, 250 Hz, 500 Hz, 1000 Hz, 2000 Hz, and 4000 Hz. Equation (1) shows the calculation method of the average sound-absorption coefficient.

(1) $$\alpha =\frac{\alpha_{125}+\alpha_{250}+\alpha_{500}+\alpha_{1000}+\alpha_{2000}+\alpha_{4000}}{6}$$

Generally, it is required that the material with a noise reduction coefficient (NRC) value greater than or equal to 0.20 is sound-absorption material [24]. NRC refers to the average value of sound-absorption coefficient at frequencies of 250 Hz, 500 Hz, 1000 Hz, and 2000 Hz. Equation (2) shows the NRC calculation method.

(2) $$\mathrm{NRC}=\frac{\alpha_{250}+\alpha_{500}+\alpha_{1000}+\alpha_{2000}}{4}\;$$

#### 2.3.2. Calculation of the Porosity

The sound absorption performance of the composite material was closely related to the porosity, which can be indirectly calculated according to the thickness and areal density of the sample [25]. The porosity of the sample was calculated using Equation (3):

(3) $$\eta \%=\left(1-\frac{G}{\rho \times \sigma }\right)\times 100$$

where η is the porosity of the sample (%), G is the surface density of the sample (g/cm^2^), *ρ* is the mixed specific gravity of the fibers (g/cm^3^), and σ is the thickness of the sample (m).

#### 2.3.3. Fractal Characterization

The two dimensional images of the sample were taken by scanning electron microscope (JEOL JEOL Ltd., Beijing, China) and the pixel size of the captured image was 1024 × 1024. Additionally, a series of pre-processing was carried out on the image using Matlab software (MATLAB 2014) programming, which made the image background consistent and the image clearer. Subsequently, the image with gray levels was converted into a binary image that could be recognized by the computer, and the box counting method was applied to calculate the fractal dimension of the sample using the Matlab program (the procedure is in Annex A) [26].

The calculation principle of the box counting dimension method is based on taking a small cube box with an edge length of ε and covering the curve graph with fractal characteristics. Some boxes are empty, some boxes contain a part of the curve, and the number of boxes that contain the curve is recorded as N(ε) [27]. Afterwards, the size of the box is shortened and N (ε) increases correspondingly. If ε is close to 0, the fractal dimension of the curve can be obtained using Equation (4):

(4) $$D=-\underset{\varepsilon \to 0}{\lim}\frac{\log N\left(\varepsilon \right)}{\log\varepsilon }$$

A straight line is obtained by fitting and mapping with the least-square method, and the slope of that straight line gives the fractal dimension [28]. For the box-counting dimension analysis of two-dimensional digital images, Matlab offers a rich visual graphic representation function and convenient programming ability [29], so Matlab was used for image processing, numerical analysis, and other operations.

## 3. Results and Discussion

### 3.1. Influence of the Technological Parameters on the Sound Absorption Coefficient

#### 3.1.1. Influence of the Mass Fraction of Poplar Seed Fibers on the Sound Absorption Coefficient

**Figure 2:**
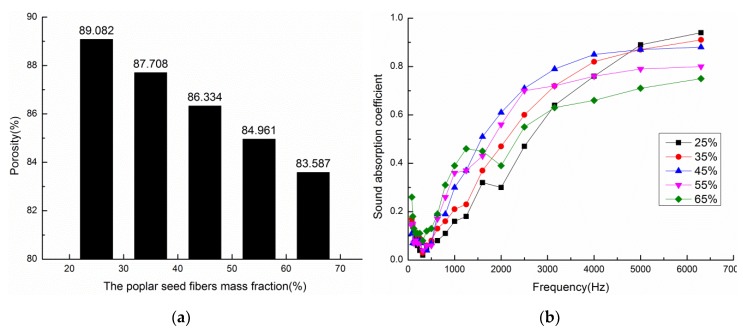
Porosities and sound absorption coefficient curves of composite materials with different mass fractions: (**a**) Porosities of composite materials (**b**) Sound absorption coefficient curves of composite materials.

Composite materials with mass fractions of poplar seed fibers of 25, 35, 45, 55, and 65% were prepared under technological conditions that consist of a volume density of 0.102 g/cm^3^ and a material thickness of 10 mm. Figure 2 shows the calculated porosities and sound absorption coefficient curves of composite materials with different mass fractions of poplar seed fibers. Figure 2a shows the porosities of composite materials. An increase in the mass fraction has a certain influence on the porosity of the composite materials, as can be seen from Figure 2a. When the mass fraction of poplar seed fibers increased from 25% to 65%, the porosity decreased from 89.08% to 83.59%. This is because the voids in a unit volume of materials containing pure poplar seed fibers (mass fraction = 100%) are larger than those in the materials containing pure PCL, so the porosity of the composite materials decreases with an increase in the mass fraction of poplar seed fibers.

Figure 2b shows the sound absorption coefficient curves of the composite materials. The mass fraction of poplar seed fibers has great effect on the sound absorption performance of the composite materials, as can be seen from Figure 2b. When the mass fraction of poplar seed fibers is 25%, the composite material obtains the maximum sound absorption coefficient, but the overall sound absorption performance of the material is relatively poor. In the frequency range of 1600 Hz to 6300 Hz, the sound absorption coefficient of the composite materials gradually increases with an increase in frequency, and the sound absorption coefficient of the composite materials tends to increase first and then decrease with an increase in the mass fraction of poplar seed fibers. The reason for this observation is that the number of micropores formed in the composite material is small when the mass fraction of poplar seed fibers is low, and the fiber agglomeration area increases due to the presence of PCL, resulting in the poor connectivity of micropores in the composite material. Therefore, the vibration of fibers and air decreases during the incident process of sound waves, and the friction between the fibers and air weakens, thus reducing the sound energy loss and poor sound absorption performance. The relative content of PCL decreases, the adhesion effect between the fibers in the composite and PCL is poor, and the connectivity of pore structure is poor when the mass fraction of poplar seed fibers is too high, which leads to the decrease of sound absorption performance. At the same time, the high fiber content cannot guarantee the molding of the material. Therefore, there is an optimal range for the mass fraction of poplar seed fibers in sound absorbing materials.

The average sound absorption coefficient and NRC of composite materials with different mass fractions can be seen in Table 1. From the table, when the mass fraction of poplar seed fibers is 45%, the average sound absorption coefficient and NRC of the composite material reach the highest, and the material is well formed. Therefore, the most suitable mass fraction of poplar seed fibers for the preparation of the composite material was set to 45%.

#### 3.1.2. Influence of the Volume Density on the Sound Absorption Coefficient

**Figure 3:**
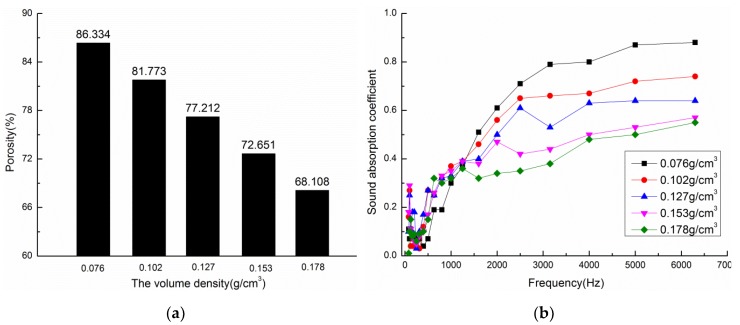
Porosities and sound absorption coefficient curves of composite materials with different densities: (**a**) Porosities of composite materials (**b**) Sound absorption coefficient curves of composite materials.

Under technological conditions consisting of a mass fraction of poplar seed fibers of 45% and a material thickness of 10 mm, composite materials with densities of 0.076, 0.102, 0.127, 0.153, and 0.178 g/cm^3^ were prepared. Figure 3 shows the calculated porosity and sound absorption coefficient curves of the composite materials for different volume densities. Figure 3a shows the porosities of composite materials. When the volume density of poplar seed fibers increased from 0.076 g/cm^3^ to 0.178 g/cm^3^, the porosity decreased from 86.33% to 68.11%, as can be seen from Figure 3a, because the change of volume density has a significant effect on the porosity of composite materials. The reason might be that, as the volume density of the material increases, the amount of fibers per unit volume in the material increases, the arrangement between the fibers and the matrix becomes denser, and fewer voids are formed, thus the porosity decreases too.

Figure 3b shows the sound absorption coefficient curves of composite materials. When the material density is 0.706 g/cm^3^, the high frequency sound absorption properties of the material is the best, and the maximum sound absorption coefficient is 0.88, but the low frequency sound absorption performance is poor, as can be seen from Figure 3b. In the same frequency range, with the increase of material density, that is, the decrease of porosity, the low frequency sound absorption performance of the composite material is obviously improved, while the high frequency sound absorption coefficient is continuously decreased, and the overall sound absorption frequency band of the material is widened. The reason for this observation is that the porosity of the composite materials with the same thicknesses will decrease and the specific flow resistance will increase with an increase of the composite material’s density, which can improve the sound absorption performance of medium and low frequencies. However, the pore size and number of micropores in the material decrease as the density of the composite material increases, which reduces the friction and vibration between air and fibers in the composite material, thus reducing the consumption of acoustic energy and the sound absorption performance. The internal structure of the material becomes more and more tight, which leads to the increase of the internal flow resistance of the composite material, the sound energy of the reflection is more, and the sound energy of the transmission is reduced, which results in a decrease in the sound absorption coefficient.

Table 2 illustrates the average sound absorption coefficient and NRC of composite materials with different material densities. From the table, when the material density is 0.102 g/cm^3^, the average sound absorption coefficient and NRC of the composite material reaches the highest, and the material is well formed, the sound absorption coefficient curve is relatively smooth as a whole, and the sound absorption frequency band is relatively wide. Therefore, the most suitable material density for the preparation of the composite material was set to 0.102 g/cm^3^.

#### 3.1.3. Influence of the Thickness on the Sound Absorption Coefficient

**Figure 4:**
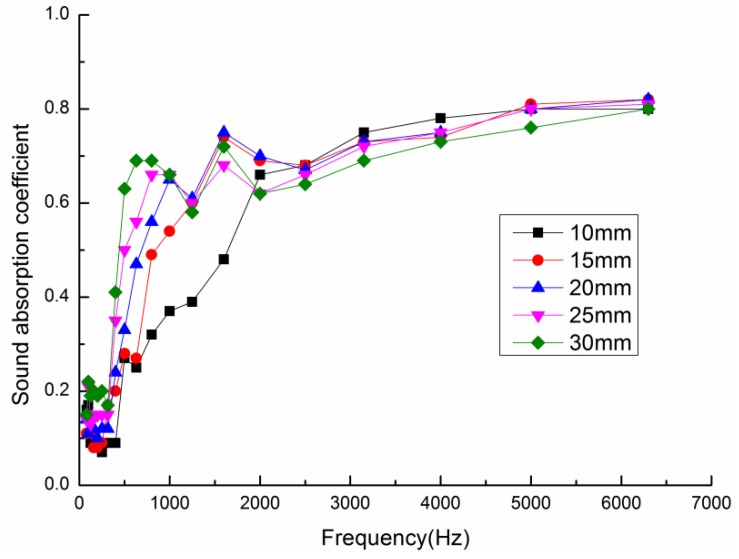
Sound absorption coefficient curves for composite materials with different thicknesses.

Composite materials with thicknesses of 10, 15, 20, 25, and 30 mm were prepared under technological conditions consisting of a volume density of 0.102 g/cm^3^ and a mass fraction of poplar seed fibers of 45%. Figure 4 shows the sound absorption coefficient curves for composite materials with different material thicknesses. 

With an increase in the thickness, the peak of the sound absorption coefficient rapidly moves in the low frequency direction, and the effective sound-absorption frequency range is expanded, as can be seen from Figure 4 [30]. At frequencies below 1500 Hz, the sound absorption coefficients increased with an increase in the thickness of the composite materials, and the amount of increase was large. At frequencies above 1500 Hz, the sound absorption coefficient curves gradually tend to be stable with an increase in the thickness. The reason for this observation is that the high frequency sound wave has a short wavelength and it is mainly absorbed by the surface of the material. The longer the wavelength of the low frequency sound wave is, the greater the distance the sound wave passes through the pores of the material, and the more it is blocked by the bending of the pores, so the low frequency sound wave is mainly absorbed by the interior of the materials [31]. Therefore, with the increase of material thickness, the low frequency sound absorption performance of the composite material obviously improves, while the high frequency sound absorption performance decreases.

Table 3 illustrates the average sound absorption coefficient and NRC of composite materials with different material thicknesses. From the table, with the increase of the thickness of the composite material, the average sound absorption coefficient, and NRC of the material have obvious improvement, and the overall sound absorption performance of the material is the best when the thickness of the material is 30 mm; therefore, the most suitable material thickness for the preparation of the composite material was set to 30 mm.

### 3.2. Fractal Characterization Results

Because the poplar seed fiber/PCL composite material is a porous material, its pore distribution is closely related to the volume density and fiber quantity of the fiber. Therefore, this paper mainly explores the relationship between fiber quantity (i.e. the influence of volume density and thickness) and the mass percentage of poplar seed fiber and PCL (i.e. the influence of the mass fraction of poplar seed fiber) and fractal dimension, and it establishes the relationship between the fractal dimension and sound absorption coefficient, so as to make the structural analysis of the material more intuitive.

#### 3.2.1. Image Acquisition of the Composite Materials

The composite materials are thick and they exhibited a large unit area. Natural light could not penetrate, so it is difficult to explore the internal structure. Therefore, scanning electron microscope (SEM) was used to obtain images of the composite materials at a magnification of 100 times. After image processing, the basal plane fiber layer was taken for pore-structure analysis. This treatment was based on the fact that the composite materials consisted of many layers of fibers with arbitrary orientation. It is assumed that most of the fiber layer aggregates were perpendicular to the propagation direction of the sound waves. Some properties (such as the sound-absorption properties) of the single layer (base plane fiber layer) can reflect the properties of the entire composite materials.

**Figure 5:**
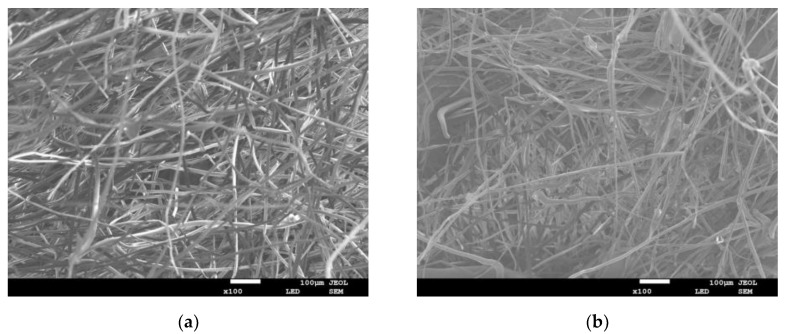

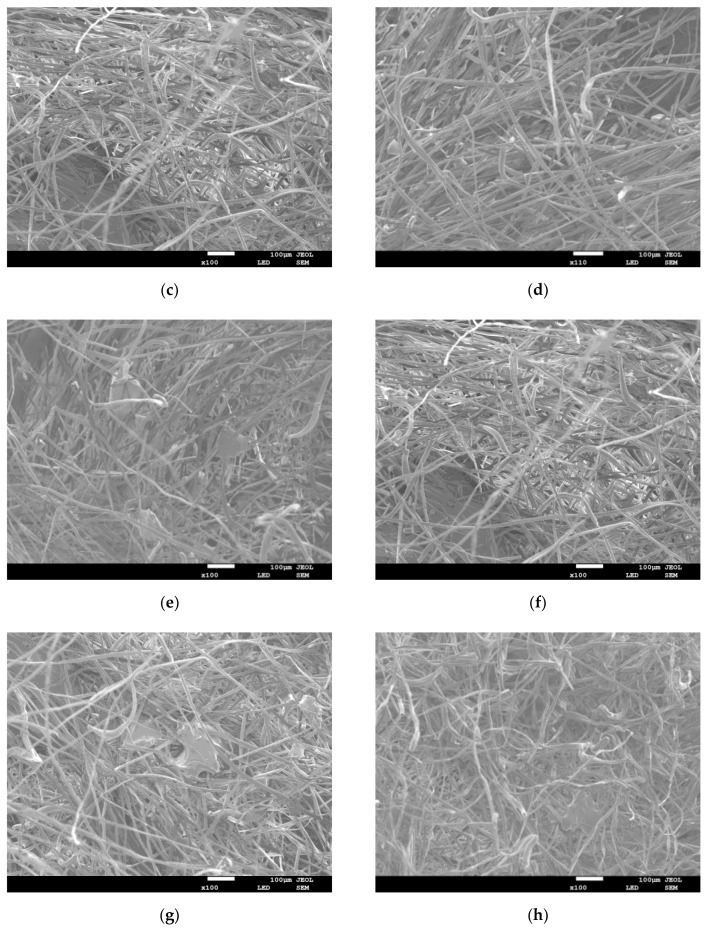

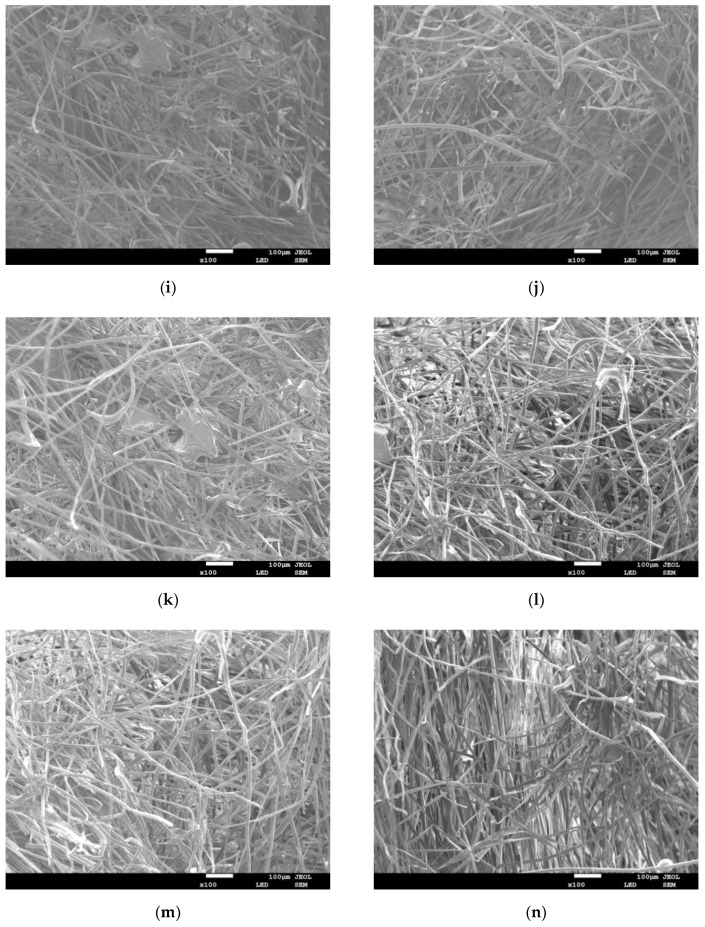

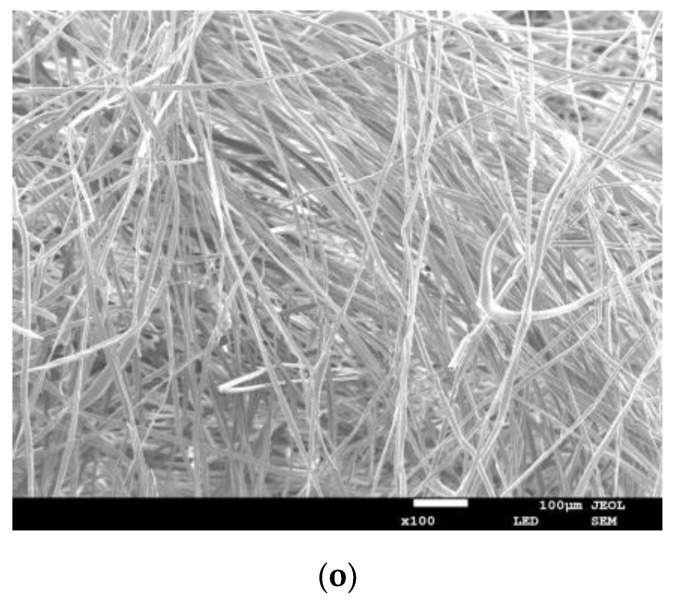
Scanning electron microscope (SEM) images of composite materials with different mass fractions, volume densities and thicknesses: (**a**) The mass fraction of poplar seed fiber is 25% (**b**) The mass fraction of poplar seed fiber is 35% (**c**) The mass fraction of poplar seed fiber is 45% (**d**) The mass fraction of poplar seed fiber is 55% (**e**) The mass fraction of poplar seed fiber is 65% (**f**) The material density is 0.076 g/cm^3^ (**g**) The material density is 0.102 g/cm^3^ (**h**) The material density is 0.172 g/cm^3^ (**i**) The material density is 0.153 g/cm^3^ (**g**) The material density is 0.178 g/cm^3^ (**k**) The Material thickness is 10 mm (**l**) The Material thickness is 15 mm (**m**) The Material thickness is 20 mm (**n**) The Material thickness is 25 mm (**o**) The Material thickness is 30 mm.

Figure 5 shows the SEM images of composite materials with different mass fractions, volume densities, and thicknesses (a–o).

#### 3.2.2. Pretreatment of the Images of the Composite Materials

A series of pretreatment are carried out on the image in order to improve the clarity and contrast of the two-dimensional composite image and make the image more conducive to accurate computer processing. Matlab software realizes the image preprocessing of each part. See Appendix A for the following procedures.

#### 3.2.3. Eliminating the Background

**Figure 6:**
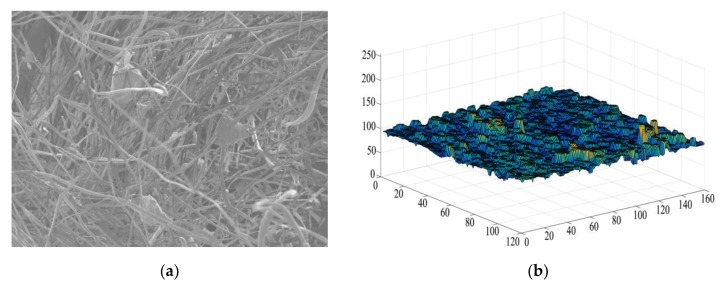

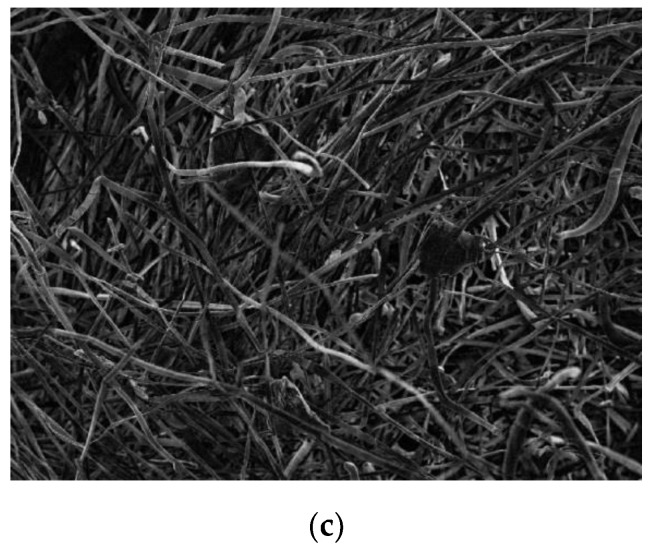
Eliminating the background: (**a**) Original image (**b**) Surface map of the background image (**c**) Two-dimensional image after eliminating the background.

The imopen function and a disc structure element with a radius of 15 were used to perform morphological opening operation on the input image to remove the objects that were not completely included in the disc, thus realizing the estimation of the background brightness, in order to eliminate the background (uneven illumination) with inconsistent brightness in the image. Subsequently, subtracted the original image from the background image to eliminate the influence of uneven background and obtain a two-dimensional image with a consistent background. Figure 6a is the original image. Figure 6b is the surface map of the background image and Figure 6c is the two-dimensional image after eliminating the background.

#### 3.2.4. Calculating a Gray Histogram and an Image After Gray Stretching

**Figure 7:**
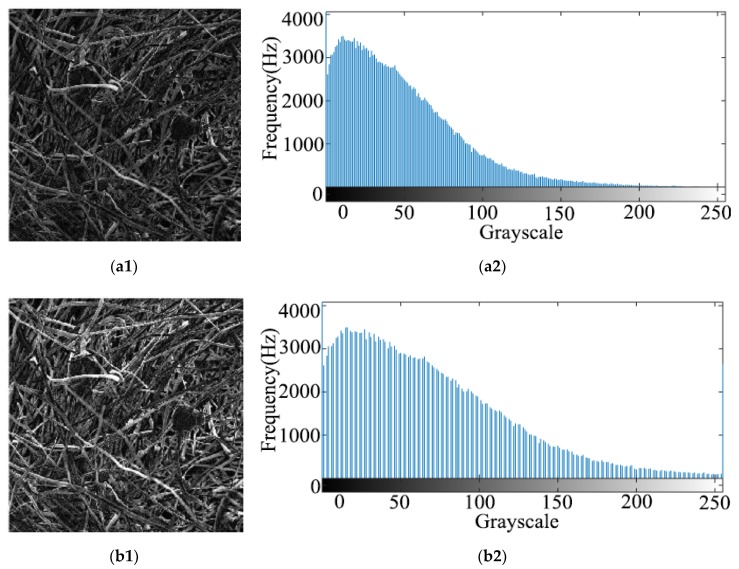
The gray scale image and grayscale histogram before and after grayscale stretching: (**a1**) Original grayscale image (**a2**) Original grayscale histogram (**b1**) Grayscale stretch effect image (**b2**) Histogram of grayscale stretched image.

The main problem of scanning electron microscope image is that the contrast of the obtained image is too low, which makes the fibers and pores confused. The pixel size of the center image was cut to 512 × 512, after gray scale stretching, the gray scale histogram with a relatively concentrated distribution was stretched to a range of 0–255, and the distribution became more uniform, which also significantly improved the contrast of the image and it was beneficial to the binarization processing of the image. Figure 7 is a gray scale diagram and a gray scale histogram before and after gray scale stretching. Figure 7(a1) is the original grayscale image, Figure 7(a2) is the original grayscale histogram, Figure 7(b1) is the grayscale stretch effect image, and Figure 7(b2) is the histogram of grayscale stretched image.

#### 3.2.5. Median Filtering of the Images

**Figure 8:**
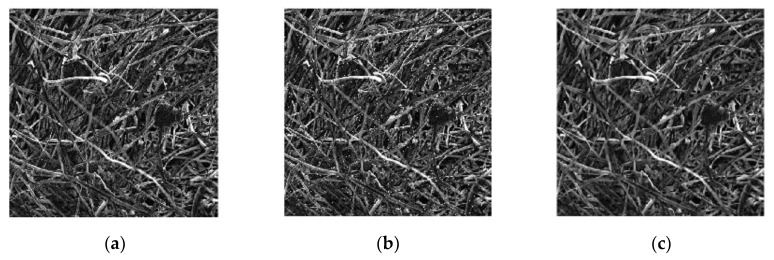
Median filtering process: (**a**) Original grayscale image (**b**) The noisy picture (**c**) Median filtered picture.

Median filtering is a kind of nonlinear signal processing technology that is based on sorting statistics theory, which can effectively suppress noise [32]. The basic principle of median filtering is to replace the value of a point in a digital image or digital sequence with the median value of each point in a neighborhood of the point, so that the surrounding pixel values are close to the real value, thus eliminating isolated noise points [33]. Figure 8 is a process diagram of median filtering processing. Figure 8a is the original grayscale image, Figure 8b is the noisy picture, and Figure 8c is the median filtered picture.

#### 3.2.6. Binarization of the Images

**Figure 9:**
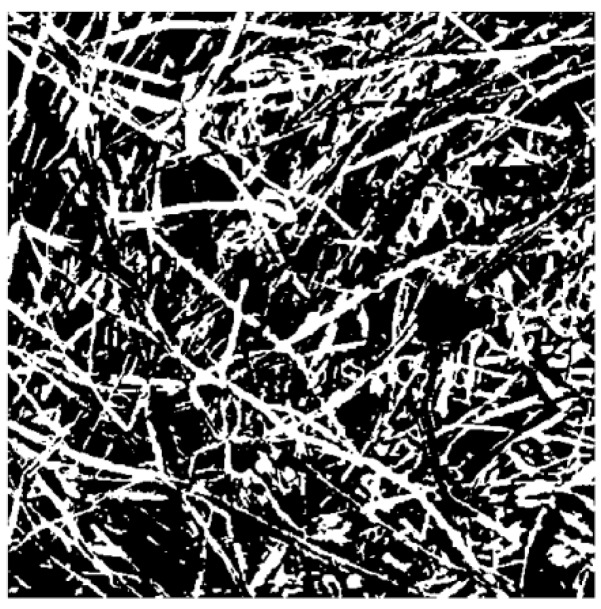
Image after binarization.

Image binarization refers to the conversion of an image with multiple gray levels into an image with two gray levels by setting a certain threshold and taking the threshold as the threshold, that is, all of the pixel points are set to black or white. There are obvious differences between the gray scale of the pore and its surrounding areas, so the image can be binarized by selecting an appropriate threshold value, changing the points with gray scale greater than or equal to the threshold value into white points, and changing the points with gray scale less than the threshold value into black points. The multi-gray level image can be changed into black and white images (two gray levels) in order to separate the pore from the fibers. Since the selection of threshold value will change the dimension size calculated, the threshold value of all two-dimensional images was determined to be 0.3 in order to increase the contrast of dimension size between different two-dimensional images. Figure 9 is a binarized image. Annex B shows the binarized images of the samples under various factors.

#### 3.2.7. Calculation of the Fractal Dimension

After the above pretreatment, the preprocessed image was taken as the input object, the box counting method was utilized to calculate the fractal dimension. The Matlab program was used to calculate the fractal dimensions of the composite materials (as shown in Appendix A), and Appendix B shows the calculation process for obtaining the fractal dimension. The fractal-dimension calculation results for composite materials with the poplar seed fiber mass fractions, volume densities and thicknesses are shown in Appendix B. 

The squares of the linear correlation coefficients are above 0.99, as can be seen from the fractal-dimension calculation results for composite materials with the poplar seed fiber mass fractions, volume densities and thicknesses, which indicates that the distribution of holes in the composite materials has fractal characteristics, and the slope of the straight line represents the fractal dimension of the holes in the samples.

#### 3.2.8. Relationship Between Fractal Dimension and Various Factors

##### Relationship between the Fractal Dimension and the Mass Fraction of Poplar Seed Fibers, Material Density and Material Thickness

**Figure 10:**
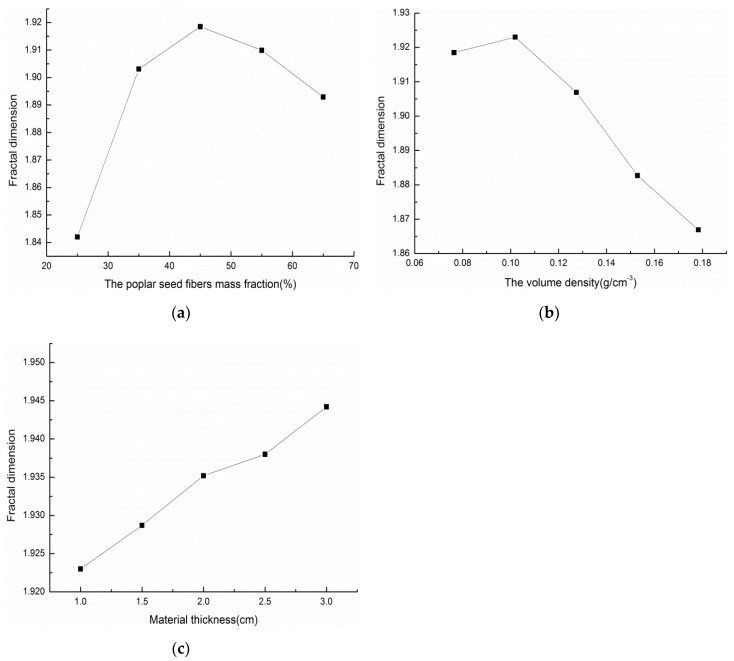
Relationship between fractal dimensions and mass fraction of poplar seed fiber, material density and material thickness: (**a**) Relation between the mass fraction of poplar seed fibers and fractal dimension. (**b**) Relationship between material density and fractal dimension (**c**) Relationship between material thickness and fractal dimension.

Figure 10 are graphs showing the relationship between the fractal dimension and mass fraction of poplar seed fiber, material density, and material thickness. Figure 10a is the relationship between the fractal dimension and the mass fraction of poplar seed fiber /PCL composite materials that were prepared under different mass fractions of poplar seed fibers. From the graph, the fractal dimension of the material increases first and then decreases with an increase in the mass fraction of poplar seed fibers, which is consistent with the change trend of the average sound absorption coefficient corresponding to the composite materials. Therefore, the fractal dimension of the composite material has certain correlation with the average sound-absorption coefficient of the material, and a high fractal dimension is obtained at the place where the average sound absorption coefficient of the material is the highest. That is, the place where the sound absorption properties of the material are the best. The fractal dimension of the material is the lowest when the mass fraction of the poplar seed fibers is 25%, mainly because under the same volume condition, the content of PCL is higher and the content of poplar seed fibers is too low, which makes the filling amount of fibers in the same space lower, resulting in a simple pore structure being formed by fiber and matrix in the material. When the mass fraction of poplar seed fibers increases, the fiber content per unit volume in the material will increase, the pore channels in the material become more complex, and the fractal dimension will increase. However, when the fiber content in the material is too high, the internal pore channels of the material will be destroyed due to a large amount of fiber accumulation, thus reducing the fractal dimension of the material.

Figure 10b is the relationship between the fractal dimension and the material density of the poplar seed fiber/PCL composite materials that were prepared under different material densities. From the graph, the fractal dimension of the material increases first and then decreases with an increase in the material density, which is consistent with the change trend of the average sound absorption coefficient corresponding to the composite material. As the density of the composite material increases, the porosity of the material will decrease, the pore structure becomes denser, the complexity of the pore channel of the material lowers, and the fractal dimension of the material will decrease correspondingly.

Figure 10c is a graph showing the relationship between the fractal dimension and the material thickness of the poplar seed fiber/PCL composite materials that were prepared under different material thicknesses. From the graph, the fractal dimension of the material will increase with an increase in the material thickness under the same conditions. The reason of this observation is that, as the thickness of the composite material increases, the length of the channel that the sound wave enters increases, the depth of the hole increases, the channel twists and turns, and the complexity increases, thus increasing the fractal dimension.

##### Relationship between Fractal Dimension and Sound Absorption Performance of Materials

**Figure 11:**
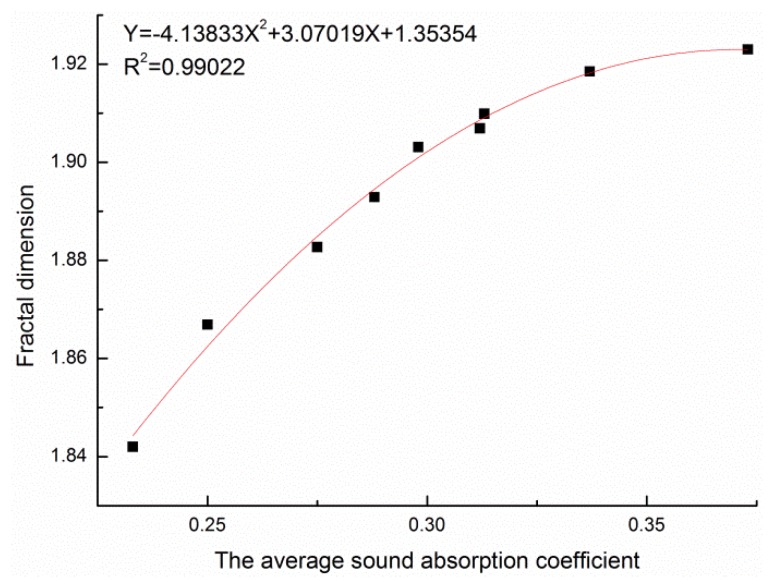
Relationship between fractal dimension and average sound absorption coefficient.

After analysis, it was found that the sound absorption performance of the poplar seed fiber/PCL composite materials had a certain correlation with fractal dimension. Therefore, the relationship curve between the average sound absorption coefficient and the fractal dimension was established by taking the experimental data under the two factors of poplar seed fiber mass fraction and material density. The fractal dimension was fitted to the average sound absorption coefficient, as shown in Figure 11.

The fitting relation was as follows:

Y = 4.13833X^2^ + 3.07019X + 1.35354 (5)

where X is the average sound absorption coefficient and Y is the fractal dimension.

The correlation coefficient of the fitting curve between the average sound absorption coefficient and the fractal dimension was 0.99, which indicated that there was a strong positive correlation between the average sound absorption coefficient and the fractal dimension. The fitting relation obtained above could be used to predict the average sound absorption coefficient of the composite material in the actual design of composite materials.

**Figure 12:**
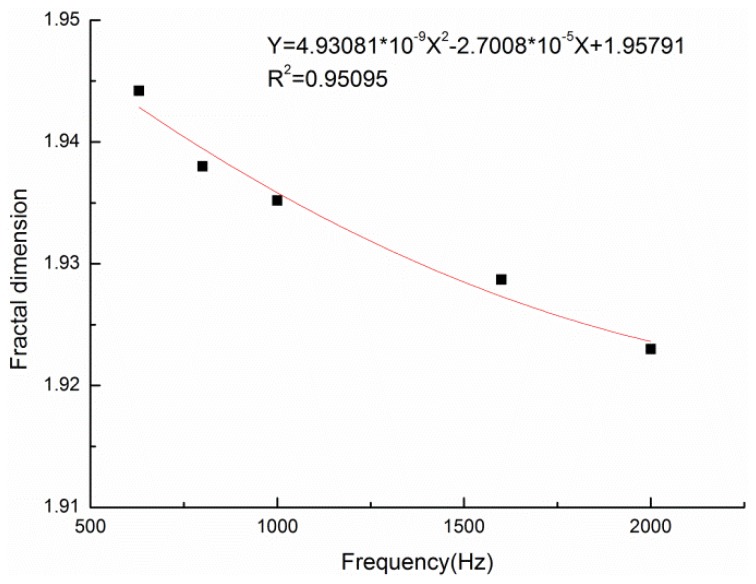
Relationship between fractal dimension and resonance absorption frequency.

The relationship between the fractal dimension and the resonant sound absorption frequency corresponding to the turning point of the sound absorption coefficient was explored as the increase of material thickness will mainly widen the sound absorption coefficient curve of the material to the low frequency, and the peak turning point of the sound absorption coefficient occurs in the whole frequency range. The fractal dimension was fitted to the resonance sound absorption frequency, as shown in Figure 12.

The fitting relation was as follows:

Y = 4.93081 × 10^−9^X^2^ − 2.7008 × 10^−5^X + 1.95791 (6)

where X is the resonance sound absorption frequency and Y is the fractal dimension.

The correlation coefficient of the fitting curve between the maximum resonance sound absorption frequency and the fractal dimension was 0.95, which indicated that there was a strong positive correlation between the maximum resonance sound absorption frequency and the fractal dimension. In the design of the actual thickness of a composite material, the fitting relation that was obtained above could be used to predict its resonance sound-absorption frequency, that is, the turning point of the sound-absorption coefficient.

## 4. Conclusions

Composite materials were prepared using poplar seed fibers and PCL as raw materials by the hot-pressing method to solve the problems related to the recycling of waste fibers. The following conclusions were drawn:

(1) The sound absorption properties of the composite materials were studied by the transfer function method. The effects of the mass fraction of poplar seed fibers, volume density, and material thickness of the composite materials on their sound absorption performance were studied. At a hot pressing temperature of 90 °C, a hot pressing pressure of 10 MPa, and a hot pressing time of 20 min., the results of single-factor experiments showed that the optimized technological conditions were: a mass fraction of poplar seed fibers of 45%, a volume density of the composite materials of 0.102 g/cm^3^, and a thickness of the composite materials of 30 mm. The sound absorption coefficient of the composite material that was prepared under the optimal process conditions was higher than 0.7, and the sound absorption frequency band was wide.
(2) Using box-counting dimension method that was based on fractal theory, the fractal dimensions of the composite materials were calculated while using the Matlab program, and the relationships between the fractal dimensions and mass fractions of poplar seed fibers, the volume densities, and thicknesses of the composite materials were analyzed. Subsequently, quantitative relationships between the average sound absorption coefficient and the fractal dimension, and between the resonant sound absorption frequency and the fractal dimension were deduced. The fractal dimension was fitted to the average sound absorption coefficient of the composite materials, and the quantitative relationship was: Y = 4.13833X^2^ + 3.07019X + 1.35354, with a correlation coefficient of 0.99. The fractal dimension was fitted to the resonance absorption frequency and the fitting curve was: Y = 4.93081 × 10^−9^X^2^−2.7008 × 10^−5^X + 1.95791, with a correlation coefficient of 0.95. By fitting the relationships between the average sound absorption coefficient and the fractal dimension, and between the resonant sound absorption frequency and the fractal dimension, a quantitative relationship of fitting curves was obtained, providing a theoretical basis and an important guide for studying and designing better sound-absorbing composite materials.

## Figures and Tables

**Table 1:** Noise reduction coefficient (NRC) and average sound absorption coefficient of composite materials that were prepared with different mass fractions.

Mass Fraction (%)	125 Hz	250 Hz	500 Hz	1000 Hz	2000 Hz	4000 Hz	NRC	Average Sound Absorption Coefficient
25	0.07	0.04	0.07	0.16	0.30	0.76	0.143	0.233
35	0.12	0.09	0.08	0.21	0.47	0.82	0.213	0.298
45	0.11	0.08	0.07	0.30	0.61	0.85	0.265	0.337
55	0.07	0.07	0.06	0.36	0.56	0.76	0.263	0.313
65	0.13	0.11	0.13	0.31	0.39	0.66	0.235	0.288

**Table 2:** NRC and average sound absorption coefficient of composite materials prepared with different densities.

Volume Density (g/cm^3^)	125 Hz	250 Hz	500 Hz	1000 Hz	2000 Hz	4000 Hz	NRC	Average Sound Absorption Coefficient
0.076	0.11	0.08	0.07	0.30	0.61	0.80	0265	0.337
0.102	0.09	0.07	0.27	0.37	0.66	0.78	0.343	0.373
0.127	0.11	0.03	0.27	0.37	0.50	0.63	0.283	0.312
0.153	0.11	0.05	0.17	0.35	0.47	0.50	0.260	0.275
0.178	0.15	0.06	0.15	0.32	0.34	0.48	0.218	0.250

**Table 3:** NRC and average sound absorption coefficient of composite materials prepared with different material thicknesses.

Material Thicknesses (mm)	125 Hz	250 Hz	500 Hz	1000 Hz	2000 Hz	4000 Hz	NRC	Average Sound Absorption Coefficient
10	0.09	0.07	0.27	0.37	0.66	0.78	0.343	0.373
15	0.11	0.09	0.28	0.54	0.69	0.74	0.400	0.408
20	0.12	0.12	0.33	0.65	0.70	0.75	0.450	0.442
25	0.13	0.15	0.50	0.66	0.62	0.75	0.483	0.468
30	0.19	0.20	0.63	0.66	0.62	0.73	0.528	0.505

**Figure A1:**
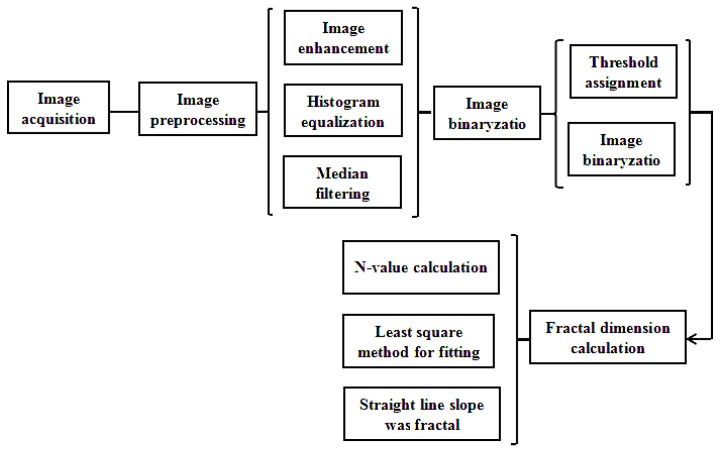
Calculation process for obtaining the fractal dimension.

**Figure A2:**
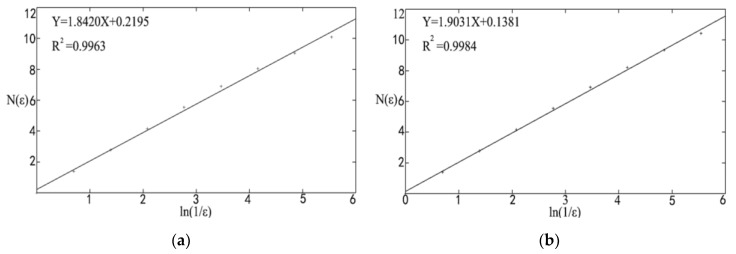

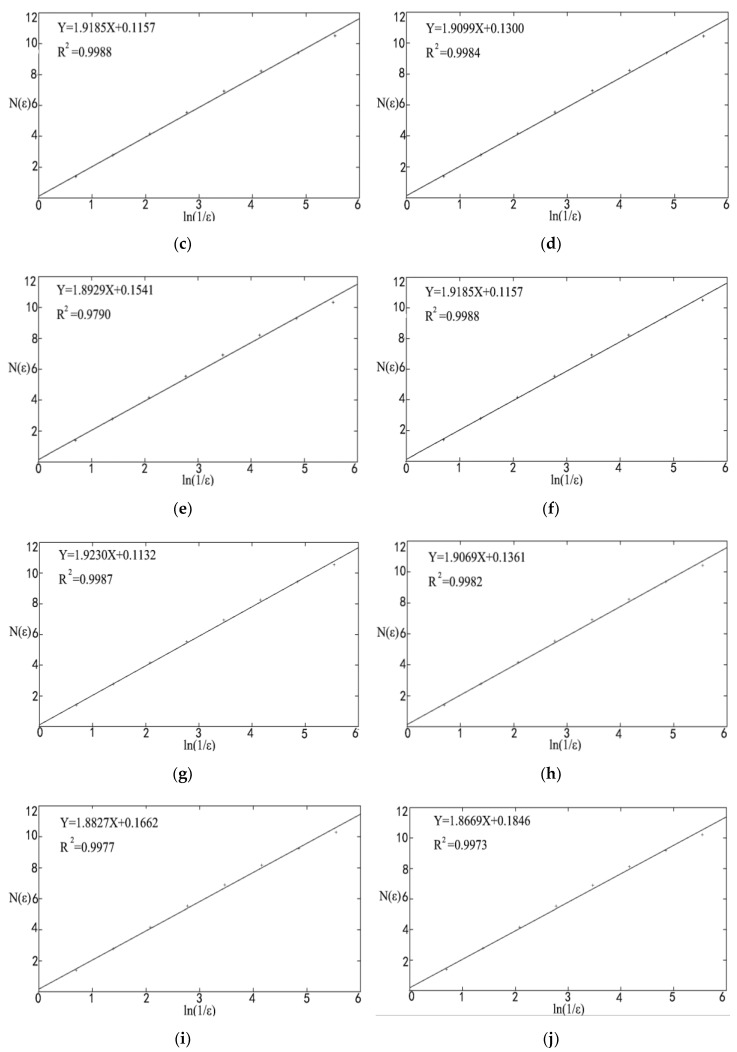

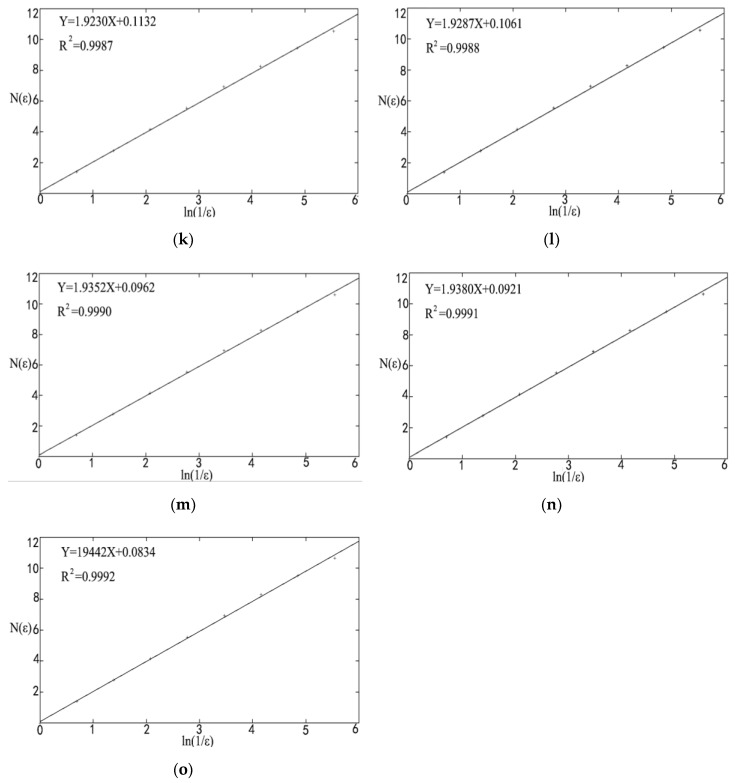
(**a**) Fractal dimension diagram with 25% fiber (**b**) Fractal dimension diagram with 35% fiber (**c**) Fractal dimension diagram with 45% fiber (**d**) Fractal dimension diagram with 55% fiber (**e**) Fractal dimension diagram with 65% fiber (**f**) Fractal dimension diagram with density of 0.076 g/cm^3^ (**g**) Fractal dimension diagram with density of 0.102 g/cm^3^ (**h**) Fractal dimension diagram with density of 0.127 g/cm^3^ (**i**) Fractal dimension diagram with density of 0.153 g/cm^3^ (**j**) Fractal dimension diagram with density of 0.178 g/cm^3^ (**k**) Fractal dimension diagram with thickness of 10 mm (**l**) Fractal dimension diagram with thickness of 15 mm. (**m**) Fractal dimension diagram with thickness of 20 mm (**n**) Fractal dimension diagram with thickness of 25 mm (**o**) Fractal dimension diagram with thickness of 30 mm

**Figure A3:**
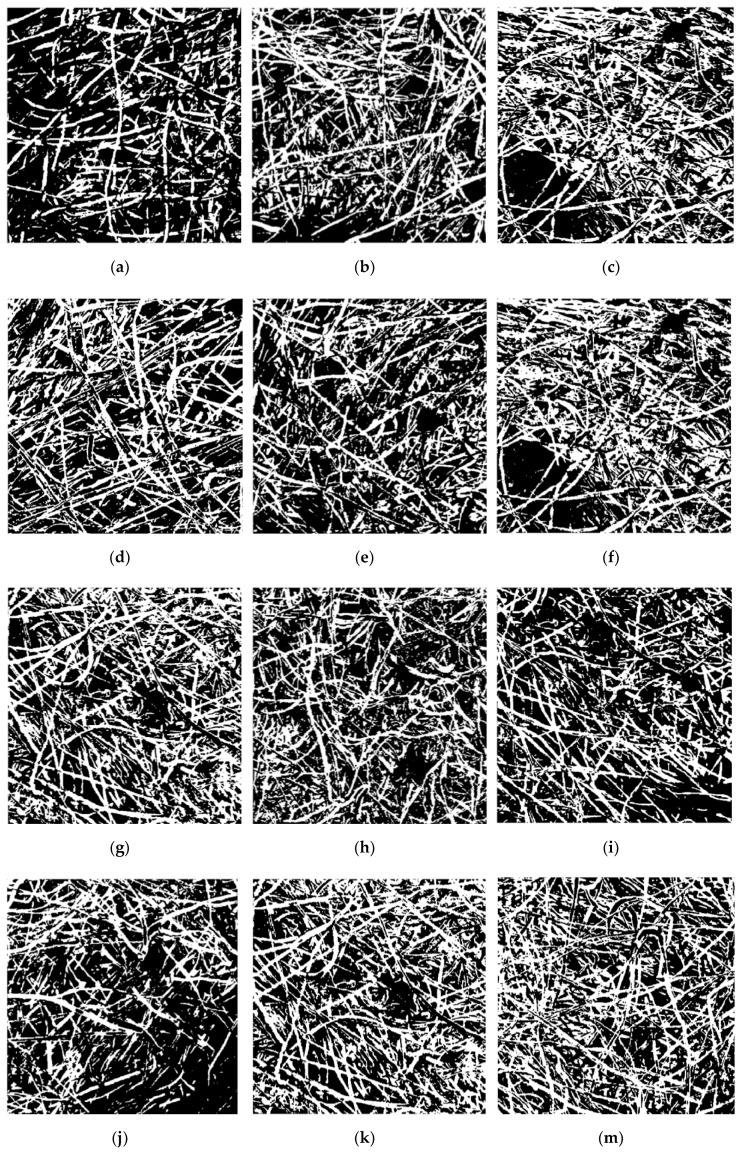

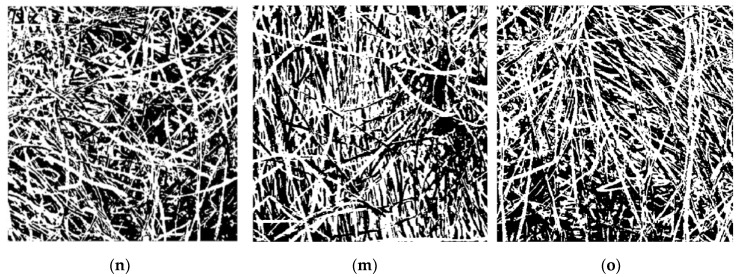
(**a**) Poplar seed fiber of 25% (**b**) Poplar seed fiber of 35% (**c**) Poplar seed fiber of 45%. (**d**) Poplar seed fiber of 55% (**e**) Poplar seed fiber of 65% (**f**) Density of 0.076 g/cm^3^ (**g**) Density of 0.102 g/cm^3^ (**h**) Density of 0.127 g/cm^3^ (**i**) Density of 0.153 g/cm^3^ (**j**) Density of 0.178 g/cm^3^ (k) Thickness of 10 mm (**l**) Thickness of 15 mm (**m**) Thickness of 20 mm (**n**) Thickness of 25 mm (**o**) Thickness of 30 mm

## Appendix A

1. The Calculation Program to Eliminate the Background

clear;close all

S1 = imread (’Original image.tif’); % Read image

imshow(S1) % Show image

background = imopen(S1, strel(’disk’,15)); % The input image I is morphologically opened with imopen function and a disc structure element with radius of 15 to remove those objects that are not completely included in the disc, thus realizing the estimation of background brightness.

imshow(background)

figure,surf(double(background(1:8:end,1:8:end))),zlim([0,255]);

set(gca,’ydir’,’reverse’);

S2 = imsubtract(S1, background);

figure,imshow(S2) % Displays the subtracted image

2. The Calculation Program of Gray Histogram and Gray Stretch Image

clear;close all

S3 = imread('S2.tif');

S4 = rgb2gray(S3); % The input image is converted into a grayscale image

Subplot (2,2,1), imshow(S4), title (' Original gray scale image');

Subplot (2,2,2), imhist(S4), title (' Original gray histogram ');

S5 = imadjust(S4); %Grayscale stretch, using function imadjust %K=imadjust (I, [low_in high_in], [low_out high_out])

Subplot (2,2,3), imshow(S5), title (' Grayscale stretch stretched diagram ');

Subplot (2,2,4), imhist(S5), title (' Histogram of grayscale stretched image ');

3. The Calculation Program of Image Median Filtering

S7 = imnoise (S5,'salt & pepper' ,0.02);

Subplot (231); imshow(S5);

Subplot (232); imshow(S7);

S8=medfilt2(S7, [3 3]);

Subplot (233);

imshow(S8);

4. The Calculation Program of Image Binarization

S9 = im2bw(S8,0.3); % Image binarization

figure,imshow(S9) % Display that processed picture

title ('Binary Image');

5. The Calculation Program of Fractal Dimension

S9 = im2bw(S8,0.3); % Image binarization

figure,imshow(S9) % Display that processed picture

title ('Binary Image');

L = size(S9,1); % Returns the number of rows for S8

if mod(log2(L),1)>0; % % mod function is a complementary function. Return the remainder of the division of two numbers. The sign of the result is the same as the divisor

  error ('The size of image must be 2^n');

end

t = log2(L); % t is 2 to the power of t, which is the number of boxes

s = 2. ^ (1:t); % s is the box size

Nr = zeros (1, t); % Create a zero matrix with 1 row and t columns

for k = 1:t;

  d = s(k);

  h = L/d;

  for m = 1:h % Cycle by row

  for n = 1:h % Cycle by column

    A = S9(d*(m-1) + [1: d], d*(n-1) + [1: d]);

    x = max (A (:));

    Nr = mx;

    Nr(k) = Nr(k)+nr;

    end

  end

  end

  r=d./s;

x = log(r);

y = log (Nr);

plot(x,y,'+');

lsline;p = polyfit(x,y,1);

xlabel('ln(1/ε)');

ylabel('N(ε)');

Dm = p;

hold on;z = y./x;

## Appendix B

1. Calculation process for obtaining the fractal dimension.

2. Fractal dimension calculation results for composite materials with different mass fractions, volume densities and thicknesses (a–o).

3. Binary images of poplar seed fiber/PCL Composite materials processed under different parameters (a–o).
